# Cultural differences in food detection

**DOI:** 10.1038/s41598-020-74388-z

**Published:** 2020-10-14

**Authors:** Wataru Sato, Krystyna Rymarczyk, Kazusa Minemoto, Sylwia Hyniewska

**Affiliations:** 1 Psychological Process Team, BZP, RIKEN, 2-2-2 Hikaridai, Seika-cho, Soraku-gun, Kyoto, 619-0288 Japan; 2 Department of Biological and Behavioral Psychology, University of Social Sciences and Humanities SWPS, Warsaw, Poland; 3 Division of Psychology and Language Sciences, University College London, London, UK

**Keywords:** Neuroscience, Psychology

## Abstract

The ability to detect food plays an indispensable role in our survival and wellbeing. Previous psychological studies have revealed that food is detected more rapidly than non-food items. However, whether the detection of food could be modulated by cultural factors remains unknown. We investigated this issue in the present study using a visual search paradigm with Polish and Japanese participants. Photographs of international fast food, domestic Japanese food, or kitchen tools were presented alongside images of non-food distractors (cars). Participants were asked to judge whether the stimuli were all identical or not. The reaction time data showed that participants from both cultures detected food more rapidly than kitchen tools. Japanese participants detected fast food more rapidly than Japanese food, whereas Polish participants did not display such differences between food types. These results suggest that rapid detection of food is universal, but is modulated by cultural experiences.

## Introduction

The detection of food is an initial and important stage in the conscious processing of food. For our ancestors, effective detection of food facilitated food intake, allowing maintenance of steady energy levels and improving the chance of survival. Food is a primary reinforcer, and appropriate detection and consumption of food plays an important role in promoting wellbeing in modern life.

Supporting this notion, several psychological studies have demonstrated that food stimuli are more rapidly detected than non-food stimuli^1–3^. For example, Sawada et al.^3^ conducted an experiment that measured reaction times (RTs) during a visual search task in which Japanese participants detected photographs of fast food, Japanese food, or kitchen tools among groups of non-food distractors (cars). The RTs for detection of food targets were shorter than those detection of kitchen tool targets, and the RTs for detection of fast food were shorter than those for detection of Japanese food. These data imply that food is rapidly detected and that conditions such as high fat content in food enhance food detection.

However, whether the detection of food could be modulated by cultural factors remains unknown. Some investigators have proposed that a nation’s food culture reflects its eating habits^4^. Each culture has its own taste preferences and beliefs about healthy foods^5^. Several cross-cultural psychological studies have shown that cultural backgrounds modulate the visual processing of food^6–8^. For example, Torrico et al.^7^ presented images of diverse food products to participants from Western and Asian backgrounds and instructed them to rate their preferences. The results showed that Western and Asian participants had higher preferences for Western and Asian food products, respectively. These findings imply that cultural factors modulate the visual processing of food, partly because we tend to prefer familiar foods.

Given the data suggesting that cultural factors modulate reactions to food, we hypothesized that detection of food could be modulated by culture. To test this hypothesis, we assessed participants from Poland and Japan using a visual search paradigm with photographs of international fast food (e.g., hamburger) or domestic Japanese food (e.g., sushi). As in a previous study^3^, participants detected food stimuli or kitchen tools (non-food targets) among displays of cars (distractors). Based on this previous study, we expected that Japanese participants would detect fast food more rapidly than Japanese food. We expected cultural differences in the detection profiles, but we could not hypothesize any specific patterns for Polish participants due to a lack of existing data. Therefore, we analyzed the data in an exploratory manner^9^ using two-tailed tests to detect any differences^10^. In addition to detection performance, we measured each participant’s preference for the food stimuli. Given the results from previous studies that tested the detection of non-food stimuli^11,12^, we expected that positive emotional reactions might enhance detection performance.

## Results

### RT

**Figure 1:**
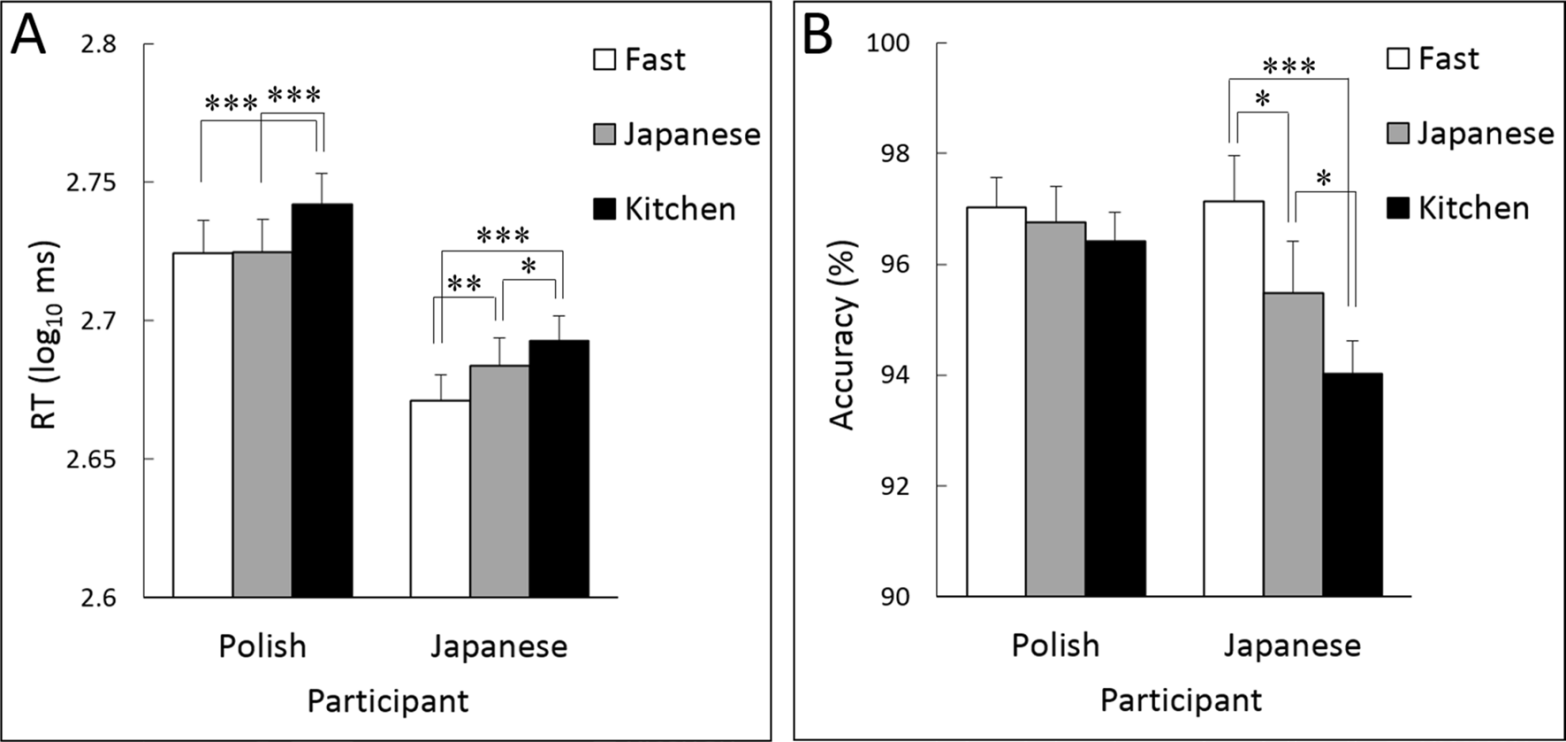
Mean (with *SE*) reaction times (RTs) (**A**) and percent accuracy (**B**) for the detection of fast food, Japanese food, and kitchen tools by Polish and Japanese participants. ***p < 0.001; **p < 0.01; *p < 0.05.

In terms of the RT to detect targets (Table 1; Fig. 1A), the 2 (culture) × 3 (stimulus type) analysis of variance (ANOVA) after log-transformation revealed significant main effects of culture (*F*(1,72) = 10.71, *p* = 0.002, *η*_*p*_^2^ = 0.13) and stimulus type (*F*(2,144) = 31.60, *p* = 0.000, *η*_*p*_^2^ = 0.31), as well as a significant interaction between these factors (*F*(2,144) = 3.21, *p* = 0.046, *η*_*p*_^2^ = 0.04).

**Table 1:** Mean (with standard error) reaction time (RT), percent accuracy, and preference ratings for fast food, Japanese food, and kitchen tools by Polish and Japanese participants.

Measure	Polish			Japanese		
	Fast	Japanese	Kitchen	Fast	Japanese	Kitchen
RT (ms)	559.3 (15.3)	537.7 (14.9)	536.7 (14.0)	497.2 (11.5)	487.5 (11.6)	472.5 (10.0)
Accuracy (%)	96.4 (0.5)	96.8 (0.7)	97.0 (0.5)	94.0 (0.8)	95.5 (0.9)	97.1 (0.6)
Preference	3.4 (0.1)	3.7 (0.1)	–	4.1 (0.1)	3.8 (0.1)	–

Follow-up analyses for the interaction showed that the simple main effects of stimulus type were significant for both Polish and Japanese groups (*F*s(2,144) = 16.09 and 18.86, respectively, *p*s = 0.000). For Polish participants, multiple comparisons showed that both fast food and Japanese food were detected more rapidly than kitchen tools (*t*s(144) = 4.97 and 4.85, respectively, *p*s = 0.000), whereas there was no significant difference between fast food and Japanese food (*t*(144) = 0.12, *p* = 0.909). For Japanese participants, both fast food and Japanese food were detected more rapidly than kitchen tools (*t*s(144) = 6.11 and 2.51, *p*s = 0.000 and 0.013, respectively), and fast food was detected more rapidly than Japanese food (*t*(144) = 3.60, *p* = 0.000).

### Accuracy

With respect to the percent accuracy of target detection (Table 1; Fig. 1B), the 2 (culture) × 3 (stimulus type) ANOVA revealed a significant main effect of stimulus type (*F*(2,144) = 7.41, *p* = 0.001, *η*_*p*_^2^ = 0.09) and a significant interaction between culture and stimulus type (*F*(2,144) = 3.38, *p* = 0.037, *η*_*p*_^2^ = 0.05).

Follow-up analyses for the interaction revealed that the simple main effect of stimulus type was significant for Japanese participants (*F*(2,144) = 10.37, *p* = 0.000) but not for Polish participants (*F*(2,144) = 0.36, *p* = 0.675). Multiple comparisons demonstrated that the differences in accuracy between fast food and kitchen tools, between fast food and Japanese food, and between Japanese food and kitchen tools were significant in Japanese participants (*t*s(144) = 4.55, 2.41, and 2.13, *p*s = 0.000, 0.017, and 0.034, respectively).

### Preference rating

**Figure 2:**
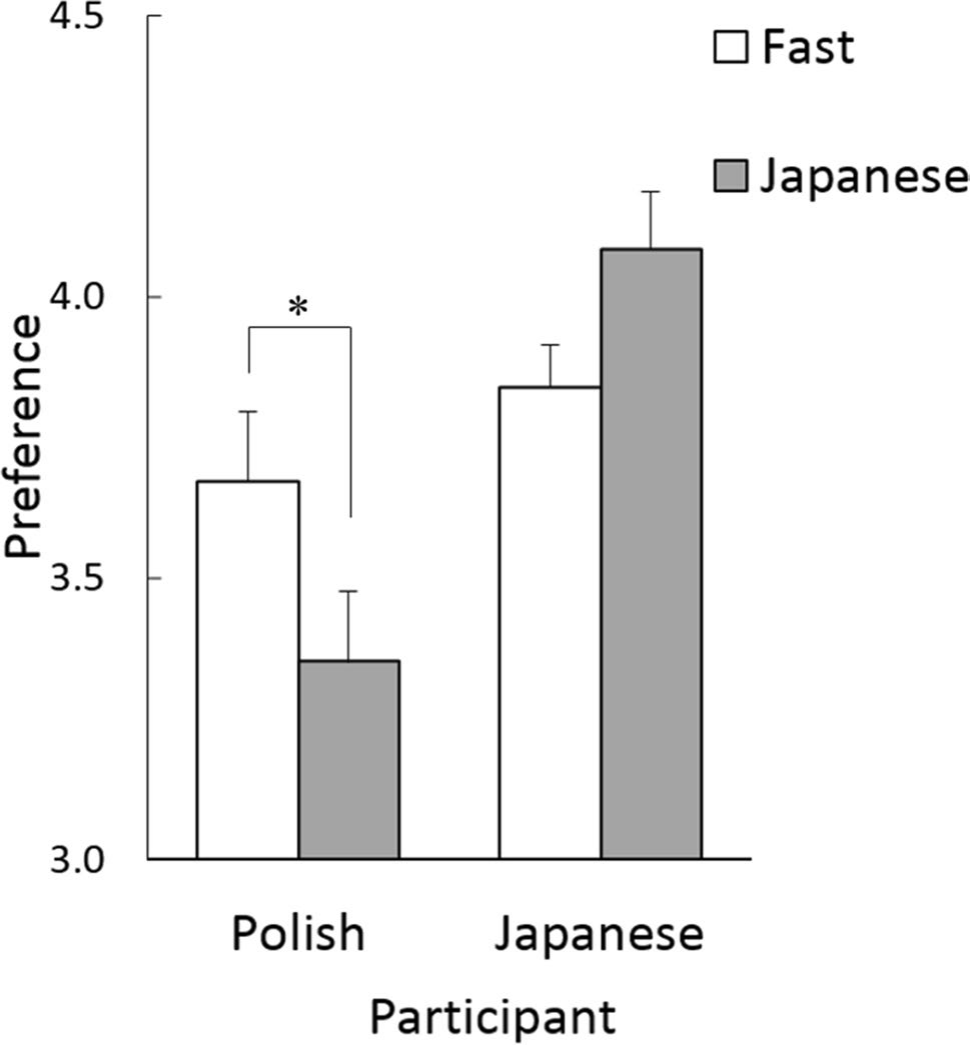
Mean (with *SE*) preference ratings for fast food and Japanese food by Polish and Japanese participants. *p < 0.05.

Regarding preference ratings for food stimuli (Table 1; Fig. 2), the 2 (culture) × 2 (food type) ANOVA revealed a significant main effect of culture (*F*(1,72) = 13.31, *p* = 0.000, *η*_*p*_^2^ = 0.16) and a significant interaction between culture and food type (*F*(1,72) = 6.00, *p* = 0.006, *η*_*p*_^2^ = 0.08).

Follow-up analyses for the interaction showed that Polish participants significantly preferred fast food to Japanese food (*F*(1,72) = 5.25, *p* = 0.025). Japanese participants had a nonsignificant tendency to prefer Japanese food to fast food (*F*(1,72) = 3.05, *p* = 0.085). Therefore, the preference ratings showed different cultural effects from those in the RT or accuracy data.

To investigate the relationship between preference ratings and detection performance, correlation coefficients were calculated between the preference rating data and RT or accuracy. The results showed no significant correlation between the preference ratings and RT (Table 2; *r*s < 0.22; Bonferroni-corrected *p*s > 0.10). In terms of accuracy, only the correlation between preference ratings and accuracy for fast food in Polish participants was significant (*r* = 0.45, Bonferroni-corrected *p* = 0.022).

**Table 2:** Correlation coefficients between preference ratings and detection performance (log_10_ reaction time [RT] and percent accuracy) for fast food and Japanese food in Polish and Japanese participants.

Detection performance	Statistic	Polish		Japanese	
		Fast	Japanese	Fast	Japanese
Log_10_ RT	*r*	0.21	− 0.09	− 0.17	− 0.02
	*p*	0.829	1.000	1.000	1.000
%Accuracy	*r*	**0.45**	0.28	0.10	0.06
	*p*	**0.022**	0.390	1.000	1.000

*P*-values were Bonferroni-corrected for each detection performance measure. Significant results are shown in bold.

## Discussion

Our investigation of detection performance in Japanese participants showed that RTs for the detection of food (both fast food and Japanese food) were shorter than RTs for the detection of kitchen tools. Accuracy, which was rather high under all conditions (> 90%), also showed better scores for both types of food items compared with kitchen tools. These results are consistent with previous findings in Japanese participants^3^. In addition, RTs in Polish participants were shorter for the detection of both fast food and Japanese food than for non-food items. These results imply that rapid food detection could be a universal phenomenon.

More interestingly, the comparison between participants’ cultures in terms of detection RT and accuracy revealed that Polish and Japanese participants displayed different patterns across food types. Specifically, whereas Japanese participants detected fast food more rapidly and accurately than Japanese food, Polish participants did not show such patterns. The results for Japanese participants are consistent with those of a previous study^3^ and may be attributable to the higher fat content of fast food compared with Japanese food. The results for Polish participants suggest that detection performance is influenced by cultural learning; for example, Japanese participants consume Japanese food often and have formed an association between Japanese food and low-fat content. For Polish participants, the lack of exposure to Japanese food and lack of association between Japanese dishes and low-fat content could explain why Polish participants did not detect fast food more rapidly than Japanese food. These results corroborate the findings of previous cross-cultural psychological studies, which showed that visual processing of food products is modulated by participants’ cultural backgrounds^6–8^. This study extends the literature and provides the first evidence that the rapid detection of food can also be modulated by participants’ cultural backgrounds.

The preference rating results showed patterns that differed from the detection performance results. Specifically, Polish participants preferred fast food to Japanese food, but they showed no comparable difference in detection between fast food and Japanese food. Correlation analyses only provided partial support for the association between preference and detection performance regarding food. Specifically, whereas the detection accuracy of fast food showed a significant correlation with preference in Polish participants, accuracy in all other conditions and RT data in all conditions did not show clear associations with preference ratings. These results suggest that, although emotional reactions may be partially related to the detection of food, as suggested in previous studies on the detection of non-food items^11,12^, other factors may have additional influences on the detection of food. Consistent with this notion, a number of previous studies have reported that other cognitive characteristics of stimuli, such as novelty or unfamiliarity, also facilitate the detection of visual stimuli^13–15^. Based on these findings, we speculate that culturally unfamiliar Japanese food was detected at a comparable level to fast food due to its novelty for Polish participants. Alternatively, because growing consumer awareness has been reported among young Polish individuals developing healthy lifestyles^16^, the perception of healthiness might have influenced Polish participants’ detection of fast food and Japanese food.

Our results have theoretical implications for the broad field of visual processing. Some theories consider the psychological mechanisms underlying visual search performance and list numerous stimulus features (e.g., motion) that facilitate detection performance^17,18^. However, there is less focus on cultural factors, such as how participants’ cultural backgrounds determine culture-specific visual guidance. Our results clearly show that detection performance for specific food stimuli in a visual search paradigm varies among participants from different cultural backgrounds. Therefore, further research is needed to investigate the effects of cultural factors on the detection of various food and non-food stimuli.

Our results also have practical implications, because understanding detection of food products among cultures is important for food product companies, particularly for globalized food trading exchanges^19^. Our data suggest that food products’ cultural novelty may boost their detection irrespective of nutritional content.

There were several limitations to the present study. First, it only included participants in two cultures and only assessed two food types. Hence, the generalizability of the findings requires further investigation. Second, this study tested only the visual search task using a few stimulus categories. Hence, the specific factors underlying detection performance remain unidentified. Although the visual search task had an advantage in that it experimentally simulates visual search in daily life, it had a disadvantage in that it incorporated the various influences of targets, distractors, and their interactions^20^. Although we used consistent distractors (i.e., cars) and controlled for basic visual confounding features (e.g., luminance), it is possible that other factors (e.g., semantic distance between food/non-food targets and distractors) may explain the detection performance. Further studies using different targets and distractors are needed to elucidate the critical factors for food detection and its cultural modulation. Alternatively, because several other studies have implemented the dot-prove paradigm (widely used to measure attentional bias^21^) and have clearly revealed more efficient attentional shift to food images, compared with non-food images^22–27^, it may be informative to use that paradigm to investigate the effect of culture on rapid visual processing of food. Finally, this study was explorative, because we could not make directional a priori predictions regarding the effects of culture on food detection due to a lack of evidence. Although the confirmatory approach plays a major role in science, it has been noted that exploratory research can play a complementary role by providing empirical evidence to generate hypotheses, which can be tested in confirmatory studies^9^. We presume that, based on findings and speculations provided in this study, future confirmatory studies may deepen the understanding of cultural influences on rapid food detection.

In conclusion, our results revealed that, while both Polish and Japanese participants showed more rapid detection of both fast food and Japanese food compared with kitchen tools, only Japanese participants detected fast food more rapidly than Japanese food. Polish participants did not show such detection performance differences across food types, possibly due to the novelty of Japanese food. These results imply that rapid detection of food is universal, but is modulated by cultural factors. Accordingly, Rozin^28^ proposed that culture and social context have a great impact on food choice, and as Montanari^29^ claimed, “the mind, shaped by culture, plays the most important role in tasting food.”

## Methods

### Participants

In all, 37 healthy Polish volunteers (27 women; mean ± *SD* age: 23.6 ± 3.1 years) and 37 healthy Japanese volunteers (25 women; mean ± *SD* age: 23.6 ± 3.1 years) participated in this study. The sample size was determined through a priori power analysis using G*Power software version 3.1.9.2^30^, assuming *α* of 0.05, power (1 – *β*) of 0.95, correlations among repeated measures of 0.5, non-spherical correction *ε* of 0.5, and effect size *f* of 0.25. The results showed that more than 72 participants were required. Participants were recruited through advertisements at the SWPS University of Social Sciences and Humanities in Poland and Kyoto University in Japan. All Polish participants lived in Poland and spoke Polish, while all Japanese participants spoke Japanese. Only Polish participants who did not regularly eat Japanese food (more than twice per week) were assessed. The Polish and Japanese participants were matched in terms of age (*t*-test, *p* > 0.10), sex (*χ*^2^-test, *p* > 0.10), and body mass index (mean ± *SD*: Polish = 21.8 ± 2.9; Japanese = 21.0 ± 2.0; *t*-test, *p* > 0.10). All had fasted for at least 3 h prior to the experiment. Their hunger levels were assessed at the beginning of the experiment using a 5-point scale ranging from 1 (hungry) to 5 (satiated); the results showed that the Polish and Japanese participants were matched for hunger levels (mean ± *SD*: Polish = 2.0 ± 0.6; Japanese = 2.1 ± 0.8; *t*-test, *p* > 0.10). No participant had visual deficiency. Written informed consent was obtained from all participants after the procedures had been amply explained. This study was approved by the Ethics Committee of Unit for Advanced Studies of the Human Mind, Kyoto University, Japan, and was conducted in accordance with institutional ethical guidelines and the Declaration of Helsinki.

### Experimental design

A repeated-measures two-factorial design was employed, with culture (Polish, Japanese) as a between-participants factor and stimulus type (fast food, Japanese food, kitchen tool) as a within-participants factor.

### Stimuli

**Figure 3:**
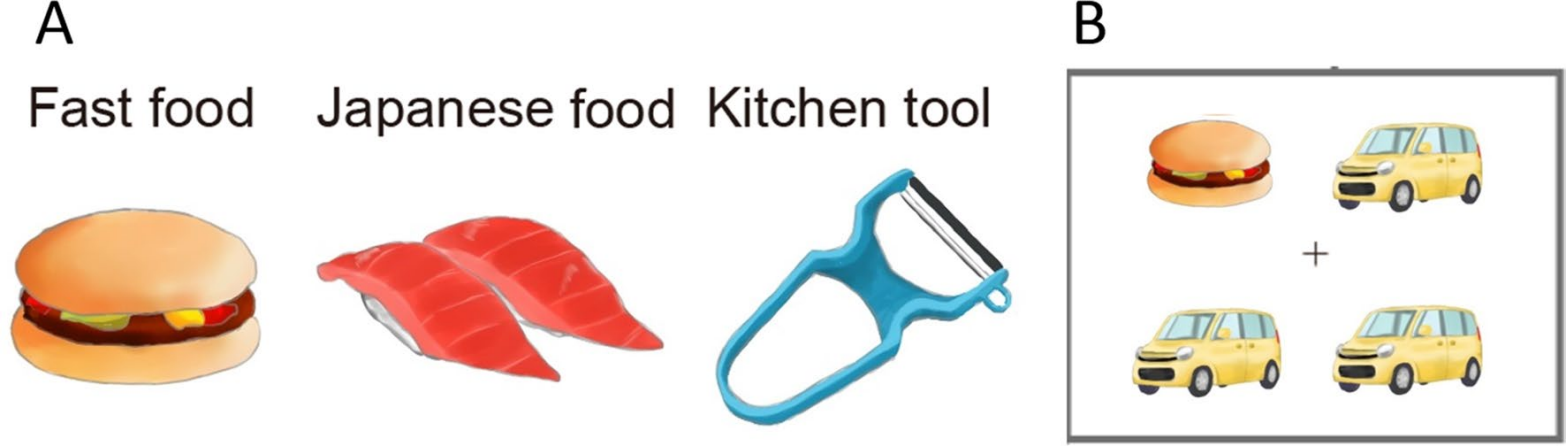
Illustrations of stimuli (**A**) and the display in the visual search task (**B**). Photographic stimuli were used in the actual experiment.

Four categories of color photographs were used: fast food, Japanese food, kitchen tool, and car. Each category had five stimuli. Therefore, 20 photos were used in total (Fig. 3A).

Three categories of food photographs were used as target stimuli: fast food (a donut, a piece of fried chicken, fried potatoes, a hamburger, and a piece of pizza), Japanese food (Japanese confection, meat and vegetable stew, sushi, noodles, and a skewer of grilled chicken), and kitchen tools (a can opener, a frying pan, a kettle, a peeler, and a scourer); car photographs were used as distractors. All photographs were selected from websites, and the backgrounds were extracted using Photoshop CS6 (Adobe, San Jose, CA, USA). The mean luminance, contrast, and RGB scores were matched among items in the fast food, Japanese food, kitchen tool, and car categories^3^. The visual angle subtended by each stimulus was 3.1 × 3.1°.

Four stimuli were displayed simultaneously in a 2 × 2 array in the visual search task. There were two sets of stimuli: the target present set, which had one food or kitchen tool stimulus and three car stimuli; and the target absent set, which had four car stimuli. The car types displayed simultaneously were all identical. The visual angle of the 2 × 2 array was 7.2 × 7.2°.

### Apparatus

Stimulus presentation was controlled by Presentation software (Neurobehavioral Systems, Berkeley, CA, USA) implemented on computers (HP Z200 SFF, Hewlett-Packard Japan, Tokyo, Japan) running Windows (Microsoft, Redmond, WA, USA). The stimuli were displayed on a 19-inch cathode ray tube monitor (HM903D-A, Iiyama, Tokyo, Japan) at a refresh rates of 100 Hz and a resolution of 1024 × 768 pixels. Responses were obtained using a response box (RB-530; Cedrus, San Pedro, CA, USA) that measures RT with 2‒3-ms resolution.

### Procedure

The study was carried out individually in soundproof rooms. Participants were seated in a comfortable position with a chin rest, while maintaining a constant distance of 57 cm from the monitor during the visual search and preference rating tasks. The total number of trials was 240, with an equal number of target present and target absent sets. Each target stimulus was presented an equal number of instances and was presented in all positions of the stimulus array. The trials were divided into four blocks of 60 trials each, and all conditions were presented randomly within each block.

In the visual search task, a 0.5 × 0.5° fixation cross was presented for 500 ms, followed by a 2 × 2 stimulus array, which was displayed until the participant responded (Fig. 3B). The interstimulus intervals varied from 500 to 800 ms. Participants were instructed to judge whether the photos in a stimulus array were identical (target absent) or whether they included one anomalous photo (target present) by pressing the appropriate key on the keypad with their right or left index finger, as quickly and accurately as possible. The key allocation was counterbalanced across participants.

After the visual search task, participants were asked to rate their preference for each food stimulus using a 5-point scale (1 = not at all to 5 = very much) written in the participant’s native language (Polish or Japanese).

### Data analysis

Data were analyzed using SPSS 16.0 J software (SPSS Japan, Tokyo, Japan). The primary performance measure in the visual search task was correct response RTs as in previous studies^3,31^. The mean RT of correct responses in target trials was calculated in each stimulus type condition for each participant, excluding data with absolute values > 3 *SD* from the mean for each participant as outliers. To satisfy normality assumptions for the subsequent analyses, the data were subjected to log_10_ transformation. The log-transformed RT data were subjected to repeated-measures ANOVA with culture (Polish, Japanese) as a between-participant factor and stimulus type (fast food, Japanese food, kitchen tool) as a within-participant factor. Mauchly’s test confirmed that the data satisfied the assumption of sphericity (*p* > 0.10). Follow-up simple main effect analyses and multiple comparisons using Ryan’s method (two-tailed) were conducted. When interactions were significant, main effects were not interpreted due to their problematic properties^32^. As the secondary performance measure, the percent accuracy was calculated and analyzed using ANOVA with the same factors. In addition, preference ratings were analyzed using repeated-measures ANOVA with culture (Polish, Japanese) as a between-participant factor and food type (fast food, Japanese food) as a within-participant factor. Correlation coefficients between the preference rating data and RT or accuracy were also calculated for each food type (fast food and Japanese food) and culture group (Polish and Japanese) for each measure (RT and accuracy). Regarding correlation coefficients, *p*-values (two-tailed) were corrected for the number of tests for each measure using Bonferroni’s method; uncorrected *p*-values (two-tailed) were multiplied by 4 with the maximum value of 1. All results were considered statistically significant when *p* < 0.05.
